# Cardiovascular Outcomes in Patients with Atrial Flutter and Oral Anticoagulation: The Predictive Role of Left Atrial Appendage Thrombus in a Long-Term, Prospective, Observational Cohort Study

**DOI:** 10.3390/jcm13247724

**Published:** 2024-12-18

**Authors:** Łukasz Turek, Marcin Sadowski, Jacek Kurzawski, Marianna Janion

**Affiliations:** The Faculty of Medicine, Jan Kochanowski University, 25-369 Kielce, Poland

**Keywords:** anticoagulation, atrial flutter, cardiovascular risk, heart failure, left atrial appendage thrombus

## Abstract

**Background/Objectives**: The risks of blood clot formation, stroke, heart failure (HF), and cardiovascular death are enhanced in individuals with atrial flutter (AFL). However, it remains unclear whether left atrial appendage thrombus (LAAT) in individuals with AFL with anticoagulation enhances the risk of cardiovascular morbidity and mortality. Thus, in the current trial, we aimed to evaluate the predictive role of LAAT for cardiovascular outcomes in individuals with AFL who were receiving anticoagulation and admitted for electrical cardioversion. **Methods**: Ninety patients were included in this prospective observational cohort study. The primary endpoint was the identification of LAAT by transesophageal echocardiographic examination. All participants were observed for a median of 2114.5 (interquartile range, 1487.5–2591) days to identify the secondary endpoints: cardiovascular death, transient ischemic attack (TIA), stroke, systemic thromboembolic complications, hospitalization due to HF, or myocardial infarction. **Results**: LAAT was identified in nine (10%) patients. No differences in cardiovascular outcomes between patients with and without LAAT were documented. However, a higher CHA_2_DS_2_-VASc score, previous myocardial infarction, and previous stroke/TIA/systemic thromboembolism were associated with significantly higher rates of hospitalization due to HF. Decreased left ventricular ejection fraction (LVEF) was associated with significantly higher rates of cardiovascular death, underscoring the significance of this marker in disease prognosis. **Conclusions**: The impact of LAAT on cardiovascular outcomes was insignificant. Higher CHA_2_DS_2_-VASc scores, previous myocardial infarction, previous stroke/TIA/systemic thromboembolism, and lower LVEF significantly affected long-term prognosis and were associated with a poor prognosis.

## 1. Introduction

The exact prevalence of atrial flutter (AFL) in the entire population remains unknown [1]. The incidence of AFL in the general population is approximately 88/100,000 person-years, and the incidence increases with age up to 317/100,000 person-years among individuals aged >50 years in the United States [2]. In 2021, there were 4.48 million new cases of atrial fibrillation (AF)/AFL worldwide, 52.5 million AF-/AFL-affected individuals, and 0.34 million AF-/AFL-related deaths; however, these data include both AF and AFL [3].

Typical AFL is a macroreentry circuit around the tricuspid annulus with the involvement of the cavotricuspid isthmus [1,4]. In a standard 12-lead electrocardiogram, a sawtooth-shaped F waveform with no isoelectric line at a rate of 250–330 beats/min and slower ventricular response is usually seen [4,5].

The risks of blood clot formation, stroke, heart failure (HF), and cardiovascular mortality are enhanced in individuals with AFL [4,6,7,8,9,10,11]. Some studies suggest a similar risk of thromboembolic events, including ischemic stroke, among individuals with AFL and those with AF, whereas other studies suggest a lower risk in individuals with AFL than in those with AF [4,6,7,12,13,14,15,16,17,18]. Oral anticoagulants are recommended for individuals with AFL at increased thromboembolic risk to prevent thromboembolism and ischemic stroke [19].

It remains unclear whether left atrial appendage thrombus (LAAT) in individuals with AFL with anticoagulation enhances the risk of cardiovascular morbidity and mortality. Therefore, to better stratify the cardiovascular risk in individuals with AFL, we aimed to investigate the relationship between the presence of LAAT and cardiovascular outcomes in individuals with AFL on chronic anticoagulation during long-term observation.

## 2. Materials and Methods

### 2.1. Study Group

We conducted an observational cohort study with patient data prospectively collected from the electronic database of the Provincial Combined Hospital in Kielce, Poland. Patients with AFL who were admitted for transesophageal echocardiography (TEE)-guided direct-current cardioversion between 31 January 2011 and 20 December 2022 were evaluated for study eligibility in accordance with the criteria in Table 1 and were consecutively enrolled in this study.

This research was conducted in line with the norms and standards of the Declaration of Helsinki. Approval was granted by the Ethics Committee of the Jan Kochanowski University of Humanities and Sciences in Kielce (protocol code: 21/2010; date of approval: 28 January 2010). Informed consent for participation in the study was obtained from all individual participants.

This research followed the Strengthening of Reporting Observational Studies in Epidemiology guidance for accurate reporting of cohort studies.

### 2.2. Anticoagulation

All individuals with anticoagulation fulfilling the following criteria were suitable for this study: treatment with vitamin K antagonist monitored with an international normalized ratio (INR) threshold level ≥ 2.0 or continuous non-vitamin K antagonist oral anticoagulants used at least 3 weeks before recruitment [20,21,22,23]. For individuals on dabigatran, a dose of 150 mg twice daily was required; however, twice-daily administration of a dose of 110 mg was also permitted in participants fulfilling ≥1 of the following clinical markers: estimated glomerular filtration rate (GFR) of 30–49 mL/min/1.73 m^2^, age ≥ 80 years, “Hypertension, Abnormal Renal/Liver Function, Stroke, Bleeding History or Predisposition, Labile INR, Elderly, Drugs/Alcohol Concomitantly” (HAS-BLED) score ≥ 3, or concomitant therapy with verapamil [21]. For individuals on rivaroxaban, therapy with 20 mg once daily was required; however, once daily administration of a dose of 15 mg was also permitted in participants fulfilling ≥1 of the following clinical markers: HAS-BLED score ≥ 3 and/or an estimated GFR of 15–49 mL/min/1.73 m^2^. For individuals on apixaban, therapy with 5 mg twice daily was required; however, therapy with 2.5 mg twice daily was allowed for individuals fulfilling ≥2 of the following: body weight ≤ 60 kg, serum creatinine level ≥ 1.5 mg/dL, or age ≥ 80 years. In the case of vitamin K antagonists, INR was checked once a week for three consecutive weeks, with all results in the expected therapeutic window. The Modification of Diet in Renal Disease equation was used for GFR calculation [24].

### 2.3. Echocardiographic Examination

Echocardiographic assessments were performed by a certified echocardiographer using ultrasound scanning equipment: GE Vivid E9 and GE Vivid E95 (GE Vingmed Ultrasound AS, Horten, Norway) with a multiplanar transesophageal ultrasound probe, following a previously described protocol [25]. Briefly, the left atrial appendage was evaluated with an appropriate depth and gain in the mid-esophageal view by a multiplanar transesophageal probe. The thrombi were recognized as an echo-dense intracardiac structure that had defined edges separate from the endocardium and was observed in two (or more) imaging planes, observed throughout diastole and systole, and separated from the pectinate muscles [26,27]. In the parasternal long-axis view using transthoracic echocardiography, the anteroposterior diameter of the left atrium was assessed on a surface perpendicular to the long axis of the ascending aorta at the end of cardiac contraction. The left ventricular ejection fraction (LVEF) was measured using the biplane Simpson’s method [28]. The outcomes of the transthoracic echocardiograms and every TEE study were recorded and are available for future studies if necessary.

### 2.4. Statistical Analyses

Percentages and frequencies were measured for categorical variables. Quartiles or means, medians, and standard deviations were calculated for continuous variables. The Shapiro–Wilk test was conducted to assess the normality of the data. When the data were normally distributed, the *t*-test was conducted; otherwise, the Mann–Whitney U test was used instead. Because the expected values were <5, Fisher’s exact test was used for the comparison of proportions. To test the differences between the continuous variables presented in Table 1, the Mann–Whitney U test was used for body mass index, age, CHA_2_DS_2_-VASc, LVEF, diastolic blood pressure, heart rate, and systolic blood pressure; for eGFR, the *t*-test for equal variances was used, and for left atrial diameter, the *t*-test for unequal variances was utilized. The hazard ratios and 95% confidence intervals were determined using univariate Cox models to identify predictors of cardiovascular mortality and hospitalization for HF. Owing to the small number of endpoints (cardiovascular death and hospitalization due to HF) achieved, multivariate Cox models were not used. If the calculated two-tailed P value was <0.05, the outcomes were considered statistically significant. Every statistical test was conducted using R (version 4.0.3, R Foundation for Statistical Computing, Vienna, Austria). A sample size calculation was not performed owing to the anticipated limited number of participants.

## 3. Results

Overall, 90 consecutive patients with AFL on oral anticoagulant therapy were included. Participants were followed up for a minimum period of one year following the TEE procedure to identify primary (LAAT presence) and secondary (death, myocardial infarction, HF hospitalization, systemic thromboembolic incidents, transient ischemic attack [TIA], and stroke) endpoints. Data were acquired from the hospital database and patients and/or family members during the first assessment after 12 months from the index hospitalization and reassessed annually.

LAAT was detected in 9 (10%) patients. A total of 38 individuals underwent direct-current cardioversion, and a catheter ablation was performed with sinus rhythm restoration in 43 patients without short-term complications. Thirty patients who had undergone direct-current cardioversion as the first option had subsequent catheter ablation.

The patients’ baseline characteristics are shown in Table 2. Individuals with LAAT had a significantly greater left atrial diameter than patients without LAAT and were more likely to have previous stroke/TIA/systemic thromboembolism with a significantly lower angiotensin II receptor blocker or angiotensin-converting-enzyme inhibitor treatment rate.

All patients were followed up for a median of 2114.5 (interquartile range, 1487.5–2591) days.

No significant differences in the occurrence of myocardial infarction, systemic thromboembolic events, stroke, TIA, HF hospitalization, and overall and cardiovascular mortality were detected (Table 3).

There was no association between a diagnosis of LAAT and the risk of cardiovascular death or hospitalization due to HF (Table 4 and Table 5). A higher CHA_2_DS_2_-VASc score, previous myocardial infarction, and previous stroke/TIA/systemic thromboembolism were associated with significantly higher rates of hospitalization due to HF (Table 4). In addition, a lower LVEF was associated with significantly higher rates of cardiovascular death (Table 5).

## 4. Discussion

The particular significance of our study lies in that the presence of LAAT in individuals with AFL with adequate anticoagulation did not increase the risk of cardiovascular death or HF-related hospitalization. We also confirmed that a higher CHA_2_DS_2_-VASc score, previous myocardial infarction, and previous stroke/TIA/systemic thromboembolism were associated with significantly higher rates of hospitalization due to HF. A decreased LVEF was the only predictor of cardiovascular death.

Although AFL increases the thromboembolic risk, few studies have focused on this association, in contrast to the numerous studies that have focused on AF. Previous echocardiographic studies have reported a thrombus in the left atrium in 0–38% of cases [11]; however, the anticoagulation status differed between the trials, and patients often did not receive any anticoagulants. For example, Huang et al. [13] conducted a retrospective study that included 114 individuals with AF and 55 individuals with AFL; individuals on chronic anticoagulant therapy and those with a prosthetic valve, congenital heart disease, moderate-to-severe mitral stenosis or regurgitation, or those with previous heart transplantation were excluded. Twelve patients (11%) in the AF group had LAAT, whereas no one in the AFL group had LAAT (*p* < 0.05). In a study by Bikkina et al. [29], the intra-atrial clot was detected in 5 of 24 individuals (21%) with AFL and in only 6 of 184 controls (3%, *p* = 0.0004). All intra-atrial thrombi were detected in men, all of which were located in the LAA. Individuals with a history of AF, those on anticoagulants, or those with a prosthetic heart valve were excluded from the trial. Parikh et al. [30] conducted a retrospective analysis of 455 individuals with AFL who underwent TEE before catheter ablation or direct-current cardioversion. Left atrial thrombi were found on TEE in 5.3% of study participants. Therapy with anticoagulant agents was noted in 62.5% (15/24) of individuals with left atrial clots and 58.9% (254/431) of individuals without left atrial clots (*p* = 0.83). Irani et al. [31] performed TEE in 47 consecutive, non-anticoagulated individuals (all men) with AFL for ≥2 days before elective cardioversion. Individuals with a history of AF, those who were on anticoagulants in the preceding 72 h, or those with mitral stenosis were excluded from the study. LAAT was present in three patients, and atrial thrombus was localized in the body of the left atrium in another two patients. In our recent trial of 69 consecutive individuals with AFL of over 48 h who were on oral anticoagulants and referred for TEE-guided direct-current cardioversion, LAAT was present in 10.1% of the study participants without a significant difference in the rate of LAAT between different oral anticoagulants [5]. In addition, frequently, AFL and AF are not considered distinctly different. For example, Kapłon-Cieślicka et al. [32] assessed the prevalence of left atrial clot in patients with AF or AFL in relation to oral anticoagulant therapy using the Left Atrial Thrombus on Transesophageal Echocardiography Registry. Among 3109 individuals referred for TEE before ablation or cardioversion, 10% were not on oral anticoagulants, 1.5% were on transients, and 88% were on chronic oral anticoagulants. Of them, a left atrial clot was present in 8.0% of individuals with AF/AFL (7.3% on chronic oral anticoagulants vs. 15% not on oral anticoagulants; *p* < 0.01). Niku et al. [33] reported on the prevalence and resolution of left atrial clots in individuals with nonvalvular AF and AFL with oral anticoagulant therapy. Among 1485 individuals with AFL or AF undergoing TEE, 117 (7.8%) had LAA thrombus or sludge (or prethrombus) on initial TEE; however, some of them did not receive any anticoagulation therapy. Myrda et al. [34] identified differences in the clinical features of individuals with AF or AFL who were referred to hospitals during the lockdown due to the coronavirus disease 2019 pandemic; they found a decrease in hospitalization of individuals with AFL or AF among individuals who were older and those with a greater number of comorbidities. Mitręga et al. [35] evaluated the effectiveness of AF/AFL-episode identification using an electrocardiographic monitoring system. Data on the rate of LAAT in individuals with AFL with anticoagulation before direct-current cardioversion are scarce, and further studies are required to elucidate this issue.

In the current study, a greater left atrial diameter and previous stroke/TIA/systemic thromboembolism were documented in the LAAT study arm; however, the left atrial diameter did not add to the risk of cardiovascular outcomes during the follow-up period. It remains unknown whether a greater left atrial diameter or previous history of stroke/TIA/systemic thromboembolism significantly increases the risk of blood clot formation in individuals with AFL and oral anticoagulation. These issues require further investigation.

In a study by Bikkina et al. [29], male sex and LVEF < 40% were predictors of left atrial blood clot formation in individuals hospitalized due to AFL. In our recent study, a prior stroke/TIA/thromboembolic event was a risk marker for predicting the presence of LAAT. However, during the 12-month follow-up period, no systemic thromboembolic events, strokes, or deaths occurred among any of the participants [5]. In a study of 455 individuals with AFL by Parikh et al. [30], participants with a CHA_2_DS_2_-VASc score of ≥2 were more prone to have both spontaneous echocardiographic contrast and left atrial clot than those with a CHA_2_DS_2_-VASc score of <2.

Although the association between AFL and LAAT is a well-documented fact, especially in patients who have not received any anticoagulant therapy, there is limited knowledge about the risk factors for thrombus formation in individuals with AFL who are already being treated with oral anticoagulation. In our study, myocardial infarction, TIA, stroke, hospitalization for HF, and cardiovascular death occurred; however, the prevalence of cardiovascular events was similar in individuals with AFL with and without LAAT, and the presence of LAAT was not a risk marker for predicting HF hospitalization or cardiovascular death. However, other clinical biomarkers, including lower LVEF as a predictor of cardiovascular death and previous myocardial infarction, previous stroke/TIA/systemic thromboembolism, and a higher CHA_2_DS_2_-VASc score as predictors of hospitalization for HF, were identified as risk factors; these are markers of disease severity in individuals with AFL on oral anticoagulation. Owing to the relationship between AFL and thromboembolic events and stroke and the frequent incidence of AF in individuals with AFL [4,6,7,8,9,10,11,19], the management of risk factors and comorbidities and the recommendations for anticoagulant therapy for individuals with AFL should reflect those for AF [4,7,19]. For individuals with AFL, anticoagulation is prescribed based on the same specific factors used for AF; however, to the best of our knowledge, there is no evidence from randomized trials focusing on the thromboembolic outcomes and benefits of anticoagulant therapy in individuals with AFL [4,5].

Based on previous studies, data on the presence of LAAT as a risk factor for cardiovascular events are limited, and further studies are warranted. Furthermore, the evidence on the predictive roles of higher CHA_2_DS_2_-VASc scores, previous myocardial infarction, previous stroke/TIA/systemic thromboembolism, and lower LVEF for cardiovascular outcomes in individuals with AFL on oral anticoagulation is limited.

Our study has some limitations. First, the number of recruited individuals in our study was relatively small. However, all consecutive eligible individuals were recruited, and several previous studies have also had relatively small sample sizes. This was a significant limitation in the calculation of the sample size. Second, patients on edoxaban were not recruited in the trial, and ultrasound contrast media were not used to improve the ultrasound visualization; therefore, overdiagnosis of blood clots of the left atrial appendage cannot be excluded. Third, the Modification of Diet in Renal Disease formula was used to calculate eGFR. However, this tool indexes GFR to a body surface area of 1.73 m^2^ and does not adjust the measurement to the current body surface area; therefore, inadequate rivaroxaban and dabigatran prescriptions may not have been excluded. Fourth, compliance with anticoagulant treatment with non-vitamin K antagonist oral anticoagulation was not verified. Nevertheless, all trial individuals demonstrated acceptable knowledge and awareness of oral anticoagulation. Fifth, we did not evaluate AFL and AF coexistence at baseline, the total duration of arrhythmia, or anticoagulation before the recruitment. Finally, we did not assess anatomical variations of the left atrial appendage or emptying velocity or the left atrial area and volume index.

## 5. Conclusions

Our findings have important clinical implications. First, we supplied strong evidence that LAAT in individuals with AFL on oral anticoagulants does not add to the risk of cardiovascular events. Additionally, data from our study support the hypothesis that a higher CHA_2_DS_2_-VASc score, previous myocardial infarction, previous stroke/TIA/systemic thromboembolism, and lower LVEF have a detrimental effect on prognosis in anticoagulated patients with AFL.

## Figures and Tables

**Table 1:** Inclusion and exclusion criteria.

**Inclusion criteria** Adult patients (age ≥ 18 years) on chronic oral anticoagulants Symptomatic and poorly tolerated atrial flutter lasting for at least 48 h **Exclusion criteria** Systolic blood pressure <90 mmHg Heart rate <60 beats/min Heart failure exacerbation Peripheral hypoperfusion Previous intracardiac thrombus Previous ablation for atrial fibrillation or atrial flutter Previous direct-current cardioversion Mitral stenosis is determined as a mitral valve area ≤1.5 cm^2^ with a mean mitral valve gradient of ≥5 mmHg Prosthetic heart valve

**Table 2:** Baseline characteristics of individuals with atrial flutter with or without left atrial appendage thrombus.

Variable	Patients with Left Atrial Appendage Thrombus (*n* = 9)	Patients Without Left Atrial Appendage Thrombus (*n* = 81)	*p*-Value
Female sex	5 (55.6%)	31 (38.3%)	0.47
Age, years	70.9 (±12.4)	67.9 (±11.1)	0.72
Body mass index, kg/m^2^	27.6 (26.0–35.8)	28.7 (27.5–32.5)	>0.99
CHA_2_DS_2_-VASc score	5.0 (3–6)	3.0 (2–4)	0.13
Heart rate, 1/min	120 (100–137)	114 (80–130)	0.72
Systolic blood pressure, mmHg	120 (110–130)	120 (110–130)	0.56
Diastolic blood pressure, mmHg	80 (70–81)	80 (70–80)	0.95
Estimated glomerular filtration rate, mL/min/1.73 m^2^	59.9 (50.2–71.4)	62.8 (54.5–73.7)	0.28
Left atrial diameter, mm	46 (45–48)	43 (39–48)	0.01 *
Left ventricular ejection fraction, %	55 (45–60)	55 (45–60)	0.56
Previous stroke/TIA/systemic thromboembolism	4 (44.4%)	4 (4.9%)	0.003 *
Arterial hypertension	7 (77.8%)	68 (84%)	0.64
Heart failure	5 (55.6%)	34 (42%)	0.49
Diabetes mellitus	1 (11.1%)	30 (37%)	0.16
Chronic obstructive pulmonary disease	0 (0%)	4 (4.9%)	>0.99
Previous myocardial infarction	1 (11.1%)	10 (12.3%)	>0.99
Peripheral artery disease or aortic plaque	0 (0%)	11 (13.6%)	0.59
Anticoagulation on admission			
⠀⠀Vitamin K antagonist	0 (0%)	10 (12.3%)	0.59
⠀⠀Rivaroxaban	1 (11.1%)	25 (30.9%)	0.44
⠀⠀Apixaban	1 (11.1%)	10 (12.3%)	>0.99
⠀⠀Dabigatran	7 (77.8%)	36 (44.4%)	0.08
Medication at discharge			
⠀⠀Vitamin K antagonist	3 (33.3%)	10 (12.3%)	0.12
⠀⠀Rivaroxaban	1 (11.1%)	25 (30.9%)	0.44
⠀⠀Apixaban	3 (33.3%)	10 (12.3%)	0.12
⠀⠀Dabigatran	2 (22.2%)	36 (44.4%)	0.29
⠀⠀Angiotensin-converting enzyme inhibitor or angiotensin II receptor blocker	2 (22.2%)	59 (72.8%)	0.004 *
⠀⠀Beta-blocker	9 (100%)	69 (85.2%)	0.6
⠀⠀Statin	6 (66.7%)	48 (59.3%)	0.74

Data are expressed as medians (interquartile ranges), means (standard deviations), or numbers (percentages), as appropriate. * Significant *p*-value. Abbreviations: CHA_2_DS_2_-VASc, scale for stroke and thromboembolic risk assessment; TIA, transient ischemic attack.

**Table 3:** Follow-up events with atrial flutter and with or without left atrial appendage thrombus.

Variable	Patients with Left Atrial Appendage Thrombus (*n* = 9)	Patients Without Left Atrial Appendage Thrombus (*n* = 81)	*p* Value
Hospitalization due to heart failure	2 (22.2%)	8 (9.9%)	0.26
Myocardial infarction	0 (0%)	1 (1.2%)	>0.99
Systemic thromboembolism	0 (0%)	0 (0%)	1
Transient ischemic attack	0 (0%)	1 (1.2%)	>0.99
Stroke	1 (11.1%)	3 (3.7%)	0.35
Cardiovascular death	2 (22.2%)	14 (17.3%)	0.66
All-cause death	2 (22.2%)	19 (23.5%)	>0.99

Data are expressed as numbers (percentages).

**Table 4:** Cox proportional hazards regression analysis with potential predictors of hospitalization due to heart failure.

Variable	Univariable HR (95% CI)	*p*-Value
Left atrial appendage thrombus	2.5 (0.52–12.11)	0.25
Male sex	0.55 (0.16–1.94)	0.35
Age, years	1.04 (0.98–1.12)	0.21
Body mass index, kg/m^2^	1.08 (0.98–1.19)	0.1
CHA_2_DS_2_-VASc score per 1	1.62 (1.12–2.35)	0.01 *
Heart rate, 1/min	1.003 (0.98–1.03)	0.78
Systolic blood pressure, mmHg	0.96 (0.9–1.02)	0.21
Diastolic blood pressure, mmHg	1.05 (0.96–1.13)	0.27
Estimated glomerular filtration rate, mL/min/1.73 m^2^	0.97 (0.93–1.02)	0.22
Left atrial diameter, mm	0.99 (0.89–1.11)	0.94
Left ventricular ejection fraction, %	0.98 (0.94–1.04)	0.56
Previous stroke/TIA/systemic thromboembolism	4.43 (1.1–17.86)	0.04 *
Arterial hypertension	^a^	
Heart failure	1.22 (0.34–4.37)	0.76
Diabetes mellitus	3.17 (0.89–11.3)	0.07
Chronic obstructive pulmonary disease	^a^	
Previous myocardial infarction	6.53 (1.45–29.34)	0.01 *
Peripheral artery disease or aortic plaque	1.07 (0.13–8.71)	0.95
Anticoagulation on admission		
⠀⠀Vitamin K antagonist	^a^	
⠀⠀Rivaroxaban	2.16 (0.57–8.2)	0.26
⠀⠀Apixaban	4.43 (0.83–23.7)	0.08
⠀⠀Dabigatran	0.63 (0.18–2.24)	0.47
Medication at discharge		
⠀⠀Vitamin K antagonist	^a^	
⠀⠀Rivaroxaban	2.16 (0.57–8.2)	0.26
⠀⠀Apixaban	3.47 (0.66–18.35)	0.14
⠀⠀Dabigatran	0.8 (0.22–2.84)	0.73
⠀⠀Angiotensin-converting enzyme inhibitor or Angiotensin II receptor blocker	0.8 (0.23–2.97)	0.77
⠀⠀Beta-blocker	0.45 (0.11–1.76)	0.25
⠀⠀Statin	0.48 (0.13–1.76)	0.27

^a^ HR was not available due to zero in one of the compared groups. * Significant *p*-value. Abbreviations: CHA_2_DS_2_-VASc, scale for stroke and thromboembolic risk assessment; HR, hazard ratio; and TIA, transient ischemic attack.

**Table 5:** Cox proportional hazards regression analysis with potential predictors of cardiovascular death.

Variable	Univariable HR (95% CI)	*p* Value
Left atrial appendage thrombus	1.45 (0.33–6.4)	0.62
Male sex	1.11 (0.4–3.1)	0.84
Age, years	1.03 (0.98–1.1)	0.18
Body mass index, kg/m^2^	0.94 (0.83–1.06)	0.31
CHA_2_DS_2_-VASc score per 1	1.15 (0.86–1.54)	0.35
Heart rate, 1/min	1.01 (0.99–1.02)	0.49
Systolic blood pressure, mmHg	1.02 (0.98–1.1)	0.31
Diastolic blood pressure, mmHg	1.03 (0.96–1.1)	0.45
Estimated glomerular filtration rate, mL/min/1.73 m^2^	0.99 (0.96–1.03)	0.67
Left atrial diameter, mm	0.94 (0.86–1.04)	0.22
Left ventricular ejection fraction, %	0.96 (0.92–0.99)	0.02 *
Previous stroke/TIA/systemic thromboembolism	^a^	
Arterial hypertension	4.43 (0.58–33.58)	0.15
Heart failure	2.25 (0.84–6.04)	0.11
Diabetes mellitus	0.68 (0.22–2.12)	0.51
Chronic obstructive pulmonary disease	3.03 (0.69–13.38)	0.14
Previous myocardial infarction	2.47 (0.68–8.88)	0.17
Peripheral artery disease or aortic plaque	0.97 (0.22–4.29)	0.97
Anticoagulation on admission		
⠀⠀Vitamin K antagonist	1.39 (0.39–4.92)	0.6
⠀⠀Rivaroxaban	0.77 (0.25–2.38)	0.64
⠀⠀Apixaban	2.62 (0.56–12.2)	0.22
⠀⠀Dabigatran	0.77 (0.29–2.08)	0.61
Medication at discharge		
⠀⠀Vitamin K antagonist	0.98 (0.28–3.47)	0.98
⠀⠀Rivaroxaban	0.77 (0.25–2.38)	0.64
⠀⠀Apixaban	1.94 (0.42–8.95)	0.39
⠀⠀Dabigatran	1.004 (0.37–2.7)	0.99
⠀⠀Angiotensin-converting enzyme inhibitor or Angiotensin II receptor blocker	2.22 (0.63–7.81)	0.21
⠀⠀Beta-blocker	1.32 (0.3–5.83)	0.71
⠀⠀Statin	0.59 (0.22–1.58)	0.3

^a^ HR was not available due to zero in one of the compared groups. * Significant *p*-value. Abbreviations: CHA_2_DS_2_-VASc, scale for stroke and thromboembolic risk assessment; HR, hazard ratio; and TIA, transient ischemic attack.

## Data Availability

Data supporting this study are available from the corresponding author, Ł.T., on reasonable request.
